# Carbon nanofiber-reinforced 3D porous aerogels for tunable low-frequency electromagnetic wave absorption

**DOI:** 10.1016/j.isci.2025.114224

**Published:** 2025-11-26

**Authors:** Xiying Shen, Pinshu Wang, Yu Chen, Ruiheng Jin, Linyu Xie, Binglin He, Xiaochi Lu, You Wen, Bin Quan

**Affiliations:** 1 School of Chemistry and Materials Science, Jiangsu Key Laboratory of New Energy Devices & Interface Science, Nanjing University of Information Science & Technology, Nanjing 210044, China; 2 College of Electronic and Optical Engineering & College of Flexible Electronics (future Technology), Nanjing University of Posts and Telecommunications, Nanjing 210023, China; 3 School of Materials Science and Engineering, Nanyang Technological University, 50 Nanyang Avenue, Singapore 639798, Singapore; 4 School of Communications and Information Engineering, Nanjing University of Posts and Telecommunications, Nanjing 210023, China; 5 School of Computer Engineering, Tongda College of Nanjing University of Posts and Telecommunications, Yangzhou 225127, China

**Keywords:** Physics, Engineering, Materials science

## Abstract

One of the main challenges electromagnetic (EM) wave absorption materials face is the difficulty to expand the effective bandwidth. Given that, here in this work, three-dimensional porous carbon-based composite aerogels were constructed by the strategy of “carbon nanofiber-assisted pyrolysis of melamine-formaldehyde resin”, which not only effectively reduces the material density but also promotes multiple scattering, establishes a continuous conductive network, introduces rich C/C hetero-interfaces, and achieves synergistic multi-mechanism loss. At a thickness of 3.5 mm, the MD-CFPC2 sample achieves a minimum reflection loss of −42.38 dB at 6.26 GHz, and the effective absorption bandwidth (RL ≤ −10 dB) covers more than 70% of the 2–18 GHz range, showing excellent low-frequency tunability. This work demonstrates the importance of design and control of pore structures in broadening the bandwidth of carbon-based EM wave absorption materials.

## Highlights

• The “carbon nanofiber-assisted pyrolysis” strategy enabled a novel material design
• The structure-property relationship enables tuning performance via carbon fiber content
• The multi-level porous and multi-interface structure delivers superior EM wave absorption

## Graphical abstract

**Figure:**
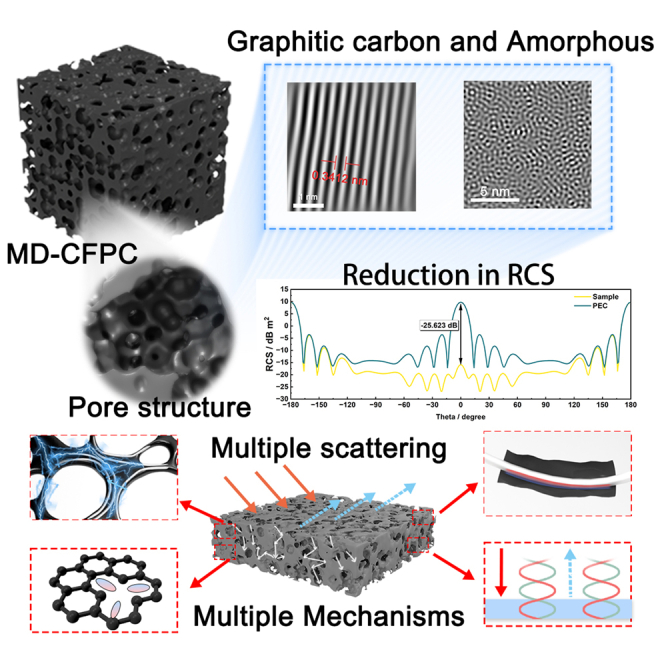

## Introduction

With the full commercialization of 5G mobile communication, Starlink low-orbit satellites, vehicle-mounted millimeter-wave radars, Wi-Fi 6/7, and drone swarm communication, the 2–18 GHz band has become the main battlefield of civilian and military electromagnetic spectrum.^1^^,^^2^^,^^3^ This frequency band carries the majority of global mobile data traffic and military radar and electronic warfare signals. On the civilian side, the extensive application of electromagnetic waves (EMW) has led to a sharp increase in power density in the environment.^4^^,^^5^ Compared to a few years ago, the risk of human bodies and equipment being exposed to strong EMW has significantly increased.^6^ This not only causes discomfort to the human body but also leads to cross-modulation and adjacent channel interference between different frequency bands due to the dense distribution of devices. On the military side, modern stealth aircraft and radars have shifted their detection frequency bands to 2-8 GHz to counter traditional X-band stealth coatings, forcing absorbing materials to expand to lower frequencies and wider bandwidths.^7^^,^^8^^,^^9^^,^^10^

This trend not only promotes the development of broadband absorbing materials and multi-frequency multi-scale materials but also poses more demands on absorbing materials.^11^^,^^12^^,^^13^^,^^14^ EMW absorbing materials must have strong EMW absorption performance, as well as meet requirements such as multi-frequency coverage,^15^^,^^16^ wideband absorption,^17^^,^^18^ ultra-thin and lightweight,^19^^,^^20^ conformability,^21^ and high-temperature resistance.^22^ This aims to improve the signal transmission quality of communication systems, reduce electromagnetic interference, and enhance the electromagnetic compatibility of equipment. Traditional ferrites and magnetic metals have drawbacks such as high density, large thickness, easy oxidation, and narrow effective bandwidth, making it difficult to meet the comprehensive requirements of “light, thin, wide, and strong”.^23^ Therefore, the development of new EMW absorbing materials has become a cutting-edge topic in functional materials research.^24^

To overcome the bottlenecks of traditional ferrites and magnetic metals, such as high density, large thickness, easy oxidation, and narrow bandwidth, many efforts have been made.^25^^,^^26^^,^^27^ Meng et al. pointed out that multi-component collaborative design is necessary to achieve lightweight, efficient, and broadband EMW absorption performance. By optimizing impedance matching with low dielectric constant materials and using carbon nanofibers (CNFs) as the framework to provide a conductive network and structural support. They constructed Ni/C@ZrO_2_ core-shell structured nanofibers, achieving a minimum reflection loss of −61.7 dB and an ultra-wideband absorption performance of 8.3 GHz.^28^ Therefore, the regulation of dielectric properties and the optimization of impedance matching are important guarantees for achieving EMW absorption performance. Han et al. retained the porous structure of sugarcane by using freeze-drying and one-step carbonization. Its unique 3D skeleton structure and high porosity promoted multiple reflections and scattering of electromagnetic waves when entering the porous structure.^29^ Which increased the propagation distance of EMW within the material and led to stronger losses. On the basis of maintaining excellent electromagnetic wave absorption performance, the construction of a porous structure can effectively reduce the density of the material while promoting multiple reflections and absorption of EMW. Carbon-based aerogels, due to their ultra-low density, high conductivity, designable microstructure, and excellent stability, are considered the most promising lightweight broadband absorbing materials.^30^^,^^31^ However, single carbon materials often have high dielectric constants and impedance mismatch, resulting in large surface reflection and weak absorption.^32^ How to achieve fine regulation of dielectric constants, enhanced interface polarization, and construction of multi-level pore structures without increasing density and thickness remains a key challenge.

Melamine-formaldehyde (MF) resin is considered a promising polymer for synthesizing porous carbon materials.^33^ Compared with other polymers, MF resin has an extremely high nitrogen content and abundant active amino groups, making it often used as a carbon and nitrogen source for preparing nitrogen-containing carbon materials. For MF, low and medium-temperature carbonization mainly forms amorphous carbon structures, which retain more heteroatoms (such as N and O), and have certain electrical conductivity. At high temperatures, the structure tends to graphitize, with an increase in sp^2^ hybridized carbon structures and enhanced electrical conductivity. Carbon fibers (CFs) materials have attracted extensive attention from researchers due to their low density, high electrical conductivity, excellent aspect ratio, good dispersibility, and strong dielectric loss capacity.^34^ However, their high dielectric constant, low magnetic permeability, and poor impedance matching still restrict their application in broadband lightweight absorption.^35^

In this study, “CFs assisted pyrolysis of MF resin” strategy was proposed. CFs serve as a physical scaffold, imposing mechanical constraints during the resin pyrolysis foaming process, forming a controllable multi-level pore network structure. The amorphous carbon structure with lower dielectric performance and highly graphitic carbon achieves optimized impedance matching. Meanwhile, the existence of the two-phase carbon leads to abundant heterojunctions, thereby enriching the electromagnetic wave absorption mechanisms. For MD-CFPC2, the minimum reflection loss (*RL*_*min*_) is −42.38 dB (6.26 GHz) at a thickness of 3.5 mm, and the effective absorption bandwidth (EAB, RL ≤ −10 dB) covers more than 70% of the 2–18 GHz range. By adjusting the mass fraction of CFs, the absorption peak position can be linearly regulated (3.7–7.2 GHz), achieving low-frequency tunable absorption. Combined with CST simulation verification, the radar cross-section (RCS) attenuation reaches −25.6 dB, demonstrating significant potential.

## Results and discussion

**Figure 1:**
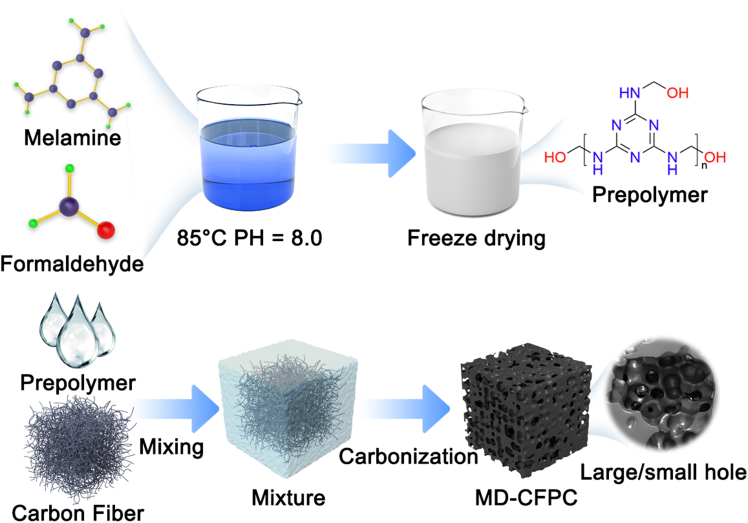
Schematic diagram of the preparation process for the MF precursor and MD-CFPC

In this study, a carbon-based EMW absorbing material with a controllable porous structure was prepared by the pyrolysis carbonization of CFs and MF resin composite system. As shown in Figure 1, an innovative CFs blending strategy was adopted to achieve dual functional regulation in the material preparation process: on the one hand, CFs served as a physical skeleton network, playing a significant role in pore size regulation during the pyrolysis foaming of MF resin. To quantitatively discuss the significance of carbon fibers during the carbonization process of MF, carbon fibers with fixed specifications were adopted, with lengths ranging from 10 μm to 20 μm and diameters from 20 nm to 50 nm. The micron-level length enables carbon fibers to form an effective network framework in the MF matrix, effectively suppressing excessive foaming and expansion during pyrolysis, thereby creating a more uniform and fine porous structure. At the same time, a high specific surface area and abundant heterogeneous interfaces are introduced, which are crucial for enhancing interface polarization and conduction loss. When the MF resin pyrolysis produced a large amount of gas, causing the matrix to expand, the dispersed CFs effectively inhibited excessive foaming through mechanical constraint effect, thereby precisely controlling the formation of a hierarchical structure with widely distributed large and small pores inside the material.^36^ This special porous structure promotes multiple scattering of electromagnetic waves.^37^ On the other hand, during the pyrolysis process, the MF resin was converted into amorphous carbon matrix, forming a continuous conductive network with high crystallinity CFs and constructing a large number of C/C heterojunctions at the interface. These heterojunction interfaces enhanced the dielectric loss capacity through interface polarization.^38^ This material system can achieve efficient dielectric regulation through component optimization. This unique “all-carbon” composite structure integrates multiple loss mechanisms. Compared with traditional ferrite absorbing materials, this carbon-based material has advantages such as high temperature resistance, oxidation resistance, and strong designability.

**Figure 2:**
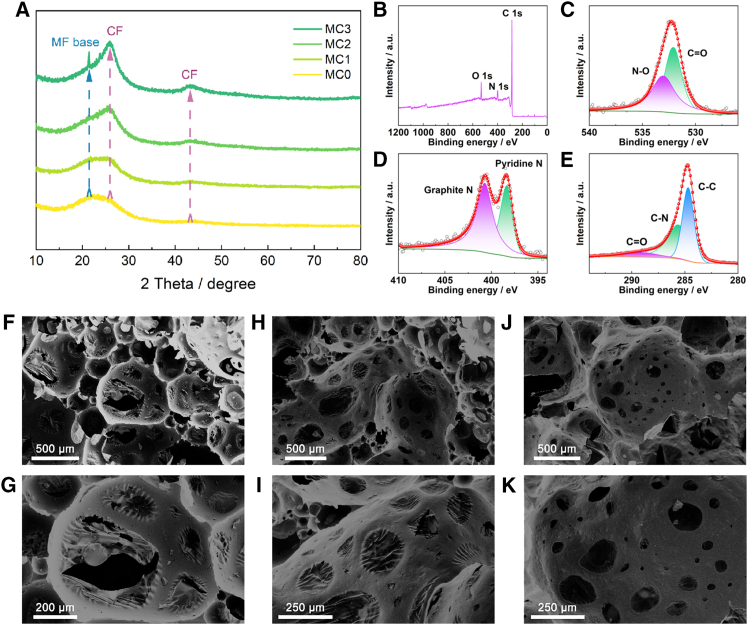
Basic characterizations of as-prepared samples (A) XRD patterns of MD-CFPC0, MD-CFPC1, MD-CFPC2, and MD-CFPC3. (B) XPS survey spectra, high-resolution XPS spectra for (C) O 1s, (D) N 1s, and (E) C 1s. SEM images of the as-prepared samples at different magnifications. (F and G) MD-CFPC0, (h, i) MD-CFPC0, and (J and K) MD-CFPC0. (Scale bars 2f, 2h, 2j: 500 *μ*m; 2g: 200 *μ*m; 2i, 2k: 250 *μ*m).

The phase composition of the product was analyzed by XRD, and the results are shown in Figure 2A. After carbonization, obvious diffraction peaks appeared at 21.40°, 25.92° and 43.18°, corresponding to the (002) and (101) diffraction peaks of amorphous carbon and graphitic carbon, confirming the successful formation of a two-component carbon composite material.^39^ Further analysis of the XRD data indicated that the increase in CFs addition amount corresponded to significant changes in the diffraction peak intensities of amorphous carbon and graphitic carbon. Specifically, with the increase of CFs, the retained graphitic carbon after carbonization significantly increased. When it increased to MD-CFPC3, an additional amorphous carbon peak appeared, which was due to the enhanced (002) crystal plane diffraction peak of low graphitic carbon at the heterojunction interface between large amounts of CFs and amorphous carbon. These changes indicate that additional CFs can significantly affect the phase composition of the final composite.

The chemical valence state and chemical bonding composition of MD-CFPC were investigated by X-ray photoelectron spectroscopy (XPS) characterization technology. The full XPS spectrum shows that the composition of MD-CFPC carbon materials is mainly dominated by C 1s, O 1s, and N 1s (Figure 2B). The fine spectrum of O 1s shows two obvious peaks at 532.1 eV and 533.0 eV, corresponding to the binding energy of C=O and N-O bonds, indicating that there are functional groups of organic pyrolysis residues in MD-CFPC (Figure 2C). The fine spectrum of N 1s can distinguish two peaks, namely Pyrrolic N and Graphite N (Figure 2D). This may be attributed to the use of nitrogen-containing precursors (MF resin) in the reaction process, and the Pyrrolic N is generated during the pyrolysis of the precursors. Pyrolysis at 700°C can result in a large amount of Pyrrolic N after the pyrolysis of melamine, providing a rich layered structure, while also containing a large number of defects and disordered regions. The presence of pyrrole N introduces significant local defects and dipole moments in the carbon lattice. These sites, as polarization centers, are prone to interact with alternating electromagnetic fields, thereby enhancing dipole polarization loss. Graphite N helps improve electron transport and effectively increases the carrier density in the carbon matrix. This promotes conduction loss by enhancing electrical conductivity and facilitating electron migration and jumping. The coexistence of pyrrole N and graphite N generates a synergistic effect. Pyrrole N enhances polarization loss, while graphite N optimizes electrical conductivity, providing a guarantee for subsequent performance optimization. The fine spectrum of C 1s can distinguish three peaks (284.7 eV, 285.6 eV, and 289.0 eV), namely C-C, C-N, and C=O bonds (Figure 2E).^40^ This is also attributed to the use of nitrogen-containing precursors (melamine resin) and the introduction of CF. The defect composition and graphitization degree of MD-CFPC carbon materials were studied by Raman spectroscopy characterization technology. The intensity of the D peak in the Raman spectrum is proportional to the defect density in the material, and the intensity of the G peak is proportional to the degree of graphitization of the material.^41^ The value of I_D_/I_G_ can be used to evaluate the defect density of the material, and a higher I_D_/I_G_ value indicates a higher defect density. As shown in Figure S2, the D peak and G peak characteristics of the two samples, MD-CFPC0 (I_D_/I_G_ = 1.044) and MD-CFPC2 (I_D_/I_G_ = 1.027), both show significant D peaks and G peaks, indicating that the samples have certain defects and a good graphitization degree. The D peak intensity of MD-CFPC0 is higher, indicating a larger defect density, while the MD-CFPC2 sample has relatively fewer defects and a slightly higher crystallinity. This is consistent with the conclusion in XPS, as the addition of CFs generates a large amount of graphitic carbon, and the precursors are the main source of defects. These defects, including vacancies, distortions, and introduced heteroatoms, play a key role in enhancing polarization loss. They act as active sites for charge accumulation, generating a large number of dipoles. Under the action of alternating electromagnetic fields, these dipoles undergo continuous reorientation and relaxation, effectively converting electromagnetic energy into thermal energy through dipole polarization, thereby making a significant contribution to the overall dielectric loss. The ID/IG ratio of the MD-CFPC2 sample was slightly lower, indicating relatively fewer defects and a high degree of graphitization, which was consistent with the increase in CFs content. A rich interface is formed between the defective amorphous carbon and graphite carbon, which is also very beneficial for interface polarization. The interaction between defective amorphous carbon and graphitic carbon creates abundant interfaces.^42^ These interfaces promote the accumulation of electrons, which, under an alternating electromagnetic field, generate dipole moments and dissipate electromagnetic energy, thereby enhancing microwave absorption performance.

The microstructure of MD-CFPC is shown in Figures 2F–2K. It can be observed that the addition of CFs has a significant impact on the structural characteristics of the obtained sample. After the introduction of CFs, the pore structure of MD-CFPC was significantly affected. Due to the mixing of CFs in MF resin, no significant fiber structure was shown on the surface of the carbonized sample. For the MD-CFPC0 sample, a large number of voids can be seen in the three-dimensional structure, and the voids are interconnected through some pores (Figures 2F and 2G). When CFs are introduced, a small amount of addition makes the pores generated inside the voids of MD-CFPC1 tend to be stable and smaller than those in MD-CFPC0 (Figures 2H and 2I). With the further increase of CF, the pores further shrink, and the pore distribution shows a dispersed state (Figures 2M and 2K). When CFs are increased to five times the initial amount, it is difficult to observe significant pores, and the surface of the voids becomes more compact (Figure S3). To quantify this process, the pore sizes in the SEM images were statistically analyzed, and the corresponding average pore size variation plots and their standard deviations were plotted. As shown in Figure S4, the pore size changes significantly with the increase of carbon fiber content. The more CFs are added, the smaller its aperture becomes, and the fluctuation of its aperture size gradually decreases. This series of changes is due to the constraint effect produced by CFs inside. In addition, thin sheets are formed within the pores of MD-CFPC0, further confirming the foaming process of MD-CFPC. This was attributed to the fact that the size of the voids has reached the critical value, and the formation of pores helps to further release the gas. In this study, a high addition of CFs inhibits the formation of three-dimensional pore structures. Therefore, by adjusting the content of CFs at a lower proportion level, the microstructure of MD-CFPC can be effectively regulated to achieve the integration of structure and function. The constructed 3D interconnected macroporous network serves as a structural backbone that facilitates efficient charge transport while simultaneously inducing extensive multiple scattering and internal reflection of incident electromagnetic waves. This structural design effectively prolongs the propagation path, enabling gradual energy attenuation across a wide frequency range.

**Figure 3:**
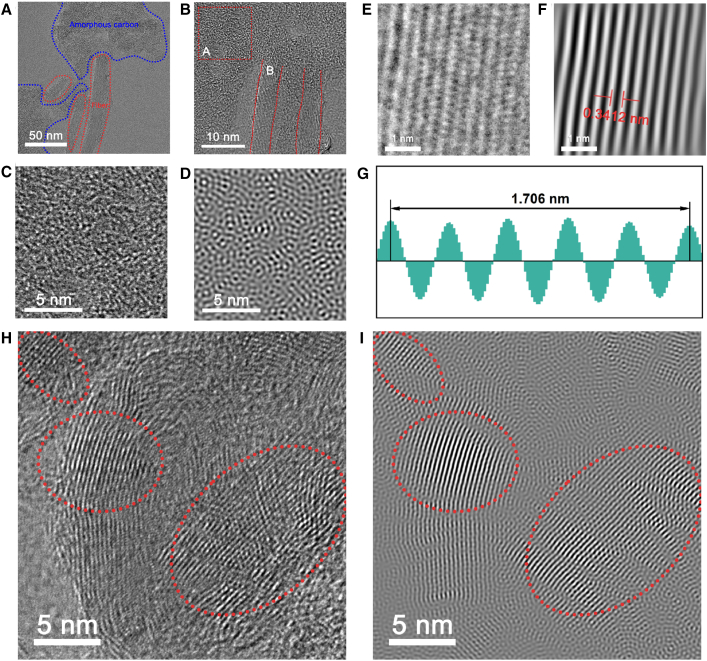
TEM image of the MD-CFPC sample (A and B) TEM image of MD-CFPC2, (C) magnified HRTEM image, (D) atomic lattice image obtained using Inverse Fast Fourier Transform (IFFT) in (C), (E) magnified HRTEM image, (F) atomic lattice image obtained using inverse fast Fourier transform (IFFT) in (E), (G) lattice spacing corresponding to (F), and (H and I) high-resolution TEM images.

To further observe the structure of MD-CFPC, TEM was used to further characterize its structure. As shown in Figures 3A and 3B, at low magnification, the sample is composed of large amorphous carbon blocks obtained by carbonizing carbon graphitic from CFs and MF resin. CFs exhibit a one-dimensional nanostructure with diameters ranging from approximately 5 to 10 nm, and are embedded in amorphous carbon, which explains the pore structure observed in the SEM characterization. It is precisely because of the restrictive effect of CFs that a unique two-component carbon structure has been constructed. Lattice spacing analysis was conducted on amorphous carbon and graphitic carbon, respectively. High-resolution TEM images showed that amorphous carbon exhibited a rather disordered lattice structure and did not have a fixed lattice spacing (Figures 3C and 3D). For graphitic carbon, they have clear lattice fringes (Figures 3E and 3F). Through the calculation of lattice spacing, a lattice spacing of 0.3412 nm can be obtained, which is consistent with the (002) crystal plane of graphitic carbon (Figure 3G). By observing the complete sheet, it can be found that there exists a regular lattice structure and an amorphous carbon lattice structure inside it, and the two are in an overlapping state (Figures 3H and 3I). Through the analysis of the lattice spacing of graphitic carbon, it can be found that the crystal plane spacing distribution is 0.3412 nm and 0.3472 nm (Figure S5). On the left, it can be observed that the positions of the significant crystal planes are consistent with the lattice spacing of the initial CF. However, in the highly overlapping parts, the lattice spacing changes, which indicates the existence of lattice stress. Meanwhile, the magnified high-resolution transmission electron microscopy (HRTEM) images show a large number of structural defects in the MD-CFPC, including lattice dislocations and point defects. The high defect level can be attributed to the pyrolysis of melamine-formaldehyde resin and the introduction of carbon fibers. Nitrogen has generated a large number of lattice defects in amorphous carbon, and carbon fibers constitute an important source of graphitic carbon. The interfacial effect of the composite reinforcement of the two has thus produced a wide variety of dipoles. When subjected to an alternating electromagnetic field, these dipoles are redirected from random arrangements to the direction of the electric field, resulting in energy dissipation.^43^ The relaxation processes of these polarized charges occur across different frequency scales, effectively dispersing the absorption capacity over a broad spectrum.

**Figure 4:**
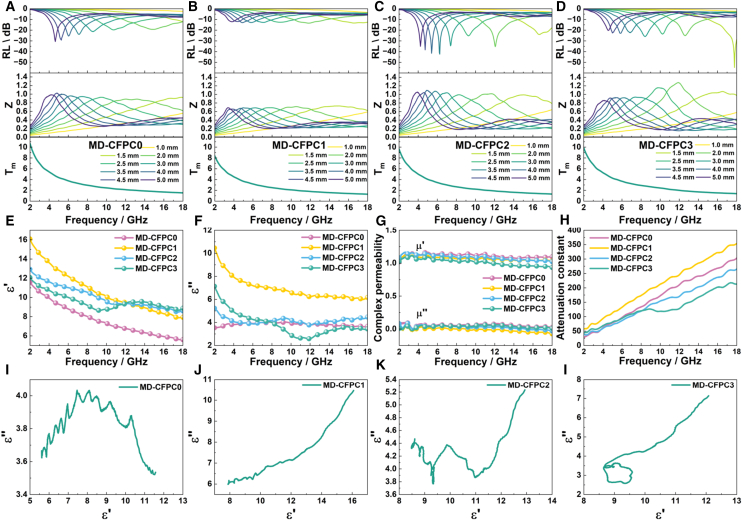
Electromagnetic wave absorption performance analysis diagram Reflection loss, impedance matching, and Tm of (A) MD-CFPC0, (B) MD-CFPC1, (C) MD-CFPC2, and (D) MD-CFPC3, (E–G) electromagnetic parameters of MD-CFPC0, MD-CFPC1, MD-CFPC2, and MD-CFPC3, attenuation constant of MD-CFPC0, MD-CFPC1, MD-CFPC2, and MD-CFPC3, Cole-Cole curve of MD-CFPC0, MD-CFPC1, MD-CFPC2, and MD-CFPC3.

EWA performance of MD-CFPC in the frequency range of 2–18 GHz was studied, as shown in Figures 4A–4D. Generally, the EWA performance mainly focuses on two key aspects: achieving the minimum reflection loss (RL) and maximizing the effective absorption bandwidth (EAB, RL ≤ −10 dB). MD-CFPC0, as the sample without added CF, already has a considerable advantage in EWA performance. At a low matching thickness of 2.0 mm, its effective absorption bandwidth reaches 6.25 GHz, and at a high matching thickness of 5.0 mm, the minimum reflection loss can reach −30.89 dB. Here, by optimizing the addition amount of CF, the EWA performance of MD-CFPC samples was effectively regulated. MD-CFPC2 significantly improved the minimum reflection without greatly reducing the effective absorption bandwidth of MD-CFPC0. Meanwhile, the EWA peak shifted to a lower frequency (at 5.0 mm, the absorption peak shifted from 3.98 GHz to 3.71 GHz). The matching thickness of MD-CFPC samples was decreased compared with that of MD-CFPC0. Therefore, the electromagnetic parameters of MD-CFPC were studied in the frequency range of 2–18 GHz (Figures 4E–4G). MD-CFPC samples have no magnetic properties, so the change in magnetic loss can be ignored. The addition of small amounts of CFs led to a significant increase in the dielectric constant of the material, while the addition of excessive amounts of CFs resulted in a decrease in the porosity and dielectric constant of the material, both of which led to a decline in EWA performance. This caused the *RL*_*min*_ and EAB of MD-CFPC1 and MD-CFPC3 to be lower than those of MD-CFPC0. This phenomenon can be attributed to the change in the distribution of CF. When small amounts of CFs are present, the inhibitory effect on the pore size is not obvious, so the carbon fibers are concentrated in the carbon skeleton shown in Figure 2F, resulting in a significant increase in the dielectric constant of the carbon skeleton. When excessive amounts of CFs are present, the inhibitory effect on the pore size is too significant, and the CFs are uniformly dispersed in the bulk, reducing the carbon fiber content per unit volume. Therefore, the dielectric constants of MD-CFPC1, MD-CFPC2, and MD-CFPC3 are all higher than that of MD-CFPC0. This indicates that adjusting the addition amounts of CFs provides an effective method for optimizing the electromagnetic properties.

The EWA performance of MD-CFPC samples is closely related to their dielectric properties, and the introduction of CFs can significantly regulate the dielectric properties of the material. Therefore, it is necessary to explore the influence of CFs introduction on the material’s performance. With the increase of CF, the conductive path is further improved, and the dielectric properties are significantly enhanced. However, an excessively high CFs content can lead to fiber accumulation, disrupting the conductive network and causing a decline in dielectric properties. An appropriate amount of carbon fiber addition helps to strike a balance between dielectric properties and pore structure. Generally, the design of high-performance absorbing materials must follow two fundamental principles: impedance matching and attenuation characteristics.^44^^,^^45^ By establishing specific boundary conditions, the reflectivity of electromagnetic waves on the material’s surface can be minimized, facilitating maximum penetration and absorption of the material. The impedance matching characteristic is usually quantified by the Z = Z_in_/Z_0_, where a value closer to 1 indicates better impedance matching performance of the absorber. The introduction of CFs successfully regulates the structure and electromagnetic properties of MD-CFPC. MD-CFPC2 has better impedance matching compared to MD-CFPC0 and MD-CFPC3. Additionally, the attenuation constant is an important parameter that describes the degree of energy attenuation of electromagnetic waves as they propagate through the medium. As shown in Figure 4H, MD-CFPC1 and MD-CFPC0 have the highest attenuation constants, but the electromagnetic wave absorption performance of MD-CFPC1 is not ideal. This is due to the impedance mismatch caused by high dielectric properties. The attenuation constant of MD-CFPC2 is reduced, but due to excellent impedance matching, its electromagnetic wave absorption performance is better. Both the attenuation constant and impedance matching performance of MD-CFPC3 are reduced, resulting in a decrease in its electromagnetic wave absorption performance. Notably, MD-CFPC1, MD-CFPC2, and MD-CFPC3 exhibit significant dispersion effects in the low-frequency range and relaxation peaks in the high-frequency range, indicating that multiple relaxations in the high-frequency range are the main source of electromagnetic wave loss mechanisms. To further clarify the dielectric loss mechanism of MD-CFPC, the Debye dielectric relaxation model is used for analysis. Based on this theory, the expression for the relative permittivity is as follows^19^:

(Equation 1) $$\varepsilon_{r}=\varepsilon_{\infty }+\frac{\varepsilon_{s}-\varepsilon_{\infty }}{1+j2\pi f\tau }=\varepsilon^{\prime }-j\varepsilon^{\prime \prime }$$

where ε_s_, ε_∞_, and τ respectively represent the permittivity of vacuum, the relative permittivity at high frequency limit, and the polarization relaxation time. Therefore, the relationship between ε′ and ε'' can be represented as follows^19^:

(Equation 2) $${\left(\varepsilon^{\prime }-\frac{\varepsilon_{s}+\varepsilon_{\infty }}{2}\right)}^{2}+{\left(\varepsilon^{\prime \prime }\right)}^{2}={\left(\frac{\varepsilon_{s}-\varepsilon_{\infty }}{2}\right)}^{2}$$

**Figure 5:**
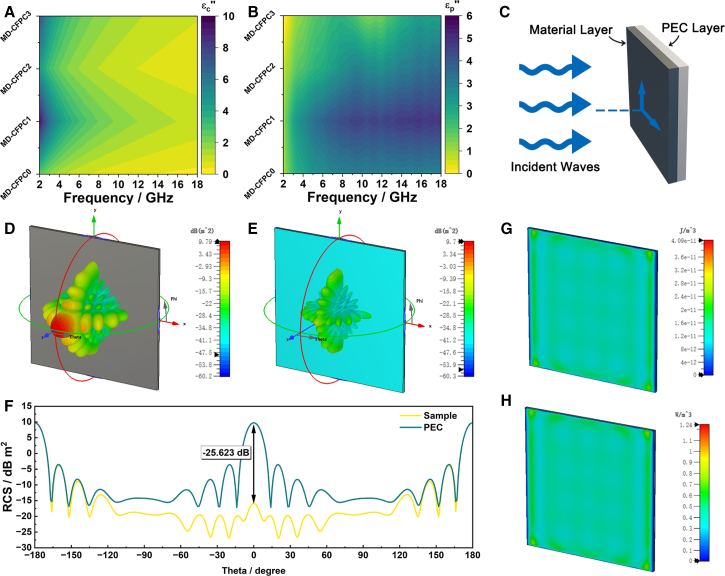
Diagram of electromagnetic wave absorption mechanism (A) conduction loss (ϵc'') and (B) polarization loss (ϵp'') of MD-CFPC, (C) radar cross-section simulation model, (D) RCS simulation results of PEC, (E) RCS simulation results of MD-CFPC2/PEC, (F) The RCS attenuation with an absorbing layer at normal incidence, (G) electric energy density, and (H) power loss density of MD-CFPC2/PEC.

Thus, the curve of ε′ and ε″ will form a semicircle, commonly known as the Cole-Cole semicircle, representing the relaxation process. As shown in Figures 4I–4L, MD-CFPC2 and MD-CFPC3 have obvious semi-circular structures, while the semi-circular structures of MD-CFPC0 and MD-CFPC1 are not obvious. This means that the addition of CFs enhances the polarization relaxation response of the material and contributes to dielectric loss in alternating electromagnetic fields. Besides multiple relaxation polarizations, the linear trend observed at the end of the curve also indicates the presence of conductivity loss. Therefore, the dielectric loss mechanism in MD-CFPC includes relaxation polarization and conductivity loss. To further dissect the dielectric loss behavior of MD-CFPC, the *ϵ''* is dissected into conduction loss (*ϵ*_*c*_*''*) and polarization loss (*ϵ*_*p*_*''*) components using Debye theory (Figures 5A and 5B).^46^ The variation trend of *ϵ*_*c*_*''* is related to the real part of the complex permittivity, indicating that the addition of carbon fibers and the change in pore size significantly affect the intrinsic conductive loss of the material. *ϵ*_*p*_*''* shows differentiated polarization losses among different samples in the 2–18 GHz range. MD-CFPC1 exhibits the strongest polarization loss, but due to its high permittivity, the impedance matching is poor. As the CF's content decreases, the planned loss also decreases. Therefore, MD-CFPC2, with its appropriate impedance matching and loss capacity, achieves more excellent electromagnetic wave absorption performance. In addition, both *ϵ*_*c*_*''* and *ϵ*_*p*_*''* values changed, along with the ratio of CF, charge transport, and dipole polarization are affected. For MD-CFPC2 and MD-CFPC3, multiple relaxation peaks are discernible from the *ϵ*_*p*_*''* curve, align with the polarization relaxation of defects and dipoles within hard carbon nanofibers, distinctly represented as Cole-Cole semicircles.

To verify the attenuation performance of MD-CFPC2, the radar scattering cross-sectional area was simulated using CST Studio Suite 2019.^47^ As shown in Figure 5C, the model adopts a double-layer plate, in which the side length of the square plate is 20 cm. The surface layer (MD-CFPC2) is an absorbing layer with a certain thickness (3.5 mm), and the backboard (perfect electrical conductors, PEC) is 1.0 mm. The double layer model plate is placed on the X-*O*-Y plane. The plane wave is incident from the positive direction of z axis to the negative direction of z axis. The electric polarization propagates along the X axis. The boundary condition sets the far-field monitor in the case of an open boundary condition in each direction. RCS simulations were conducted in two steps: PEC surfaces and surfaces with EM absorbing materials, with the results presented in Figures 5D and 5E. It is evident that the double-layer structure coated with electromagnetic EM absorbing material exhibits a significant reduction in RCS values. As exhibited in Figure 5F, compared with the single-layer PEC without the addition of MD-CFPC2, the RCS value of MD-CFPC2/PEC has an attenuation of −25.623 dB when incident vertically. Within the scattering range of -90-90°, its RCS value is always less than that of the PEC plate, indicating that this material has a significant effect in absorbing radar waves. At the same time, the Power loss density and electric energy density of the model were calculated, as shown in Figures 5G and 5H. The distributions of the two exhibit a high degree of similarity. The introduced CFs play dual roles. They substantially enhance the overall electrical conductivity and charge transport network, thereby optimizing conductive loss. Simultaneously, they suppress over-expansion of the foam during pyrolysis, resulting in a more refined and uniform distribution of pores and interfaces. This suppression is crucial for achieving improved impedance matching and a more efficient wideband absorption response.

**Figure 6:**
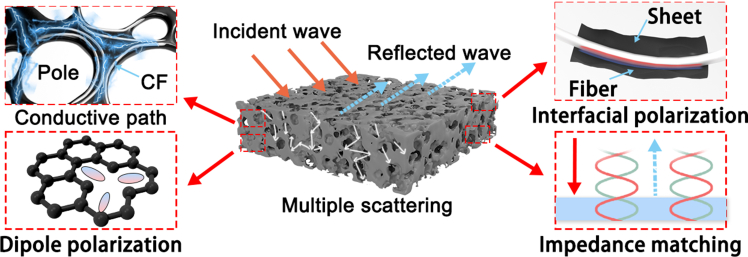
Schematic of the EMW absorption mechanisms of MD-CFPC

In this study, by precisely regulating the mass ratio of CFs to amine resin and the pyrolysis temperature, the foaming behavior and pore structure of the material were directly controlled, avoiding high-temperature graphitization and retaining nitrogen elements, thereby laying the foundation for its tunable dielectric properties. By combining solvent-assisted pyrolysis with controllable drying processes, uniform dispersion of CFs was ensured to form a continuous network and uniform interface. Ultimately, the structure-activity relationship between experimental variables and material properties was verified through various characterization techniques. The research results demonstrated that the composite of CFs and MF resin precursors effectively regulated their electromagnetic properties and porosity. The pyrolysis of the MF resin precursors generated a large number of defects, and the introduction of CFs enriched the internal interfaces, significantly enhancing the attenuation characteristics of EMW. Moreover, the unique three-dimensional network structure increased the conductivity loss and optimized the impedance matching characteristics, thereby delivering outstanding EWA performance. The EMW absorption performance of the MD-CFPC2 composite was benchmarked against other CF based absorbers, as summarized in Table S1. While numerous composites report exceptional absorption depths, their optimal performances are predominantly concentrated at higher frequencies. In contrast, the MD-CFPC2 absorber developed in this work achieves its peak performance (*RL*_*min*_ = −42.38 dB) at a significantly lower frequency of 6.26 GHz. As shown in Figure 6, which is achieved through the combined effect of multiple mechanisms: CFs act as a physical skeleton to restrict the foaming of the resin during pyrolysis, forming a multi-level pore structure and enhancing the multiple scattering of EMW; simultaneously, CFs and pyrolyzed carbon form a large number of C/C heterointerfaces, inducing the cooperative effect of interface polarization and defect dipoles, which significantly enhances the dielectric loss. Additionally, appropriate amounts of CFs optimize the conductive network and impedance matching, achieving efficient synergy between conductive loss and polarization loss, ultimately achieving excellent electromagnetic wave absorption performance in the low-frequency region. These findings provide insights for the development of high-performance absorbers for low-frequency EMW.

### Limitations of the study

This study constructed three-dimensional porous carbon-based aerogels through a carbon nanofiber-assisted melamine-formaldehyde resin pyrolysis strategy. The feasibility of industrial-scale production has not yet been explored. Despite the above-mentioned limitation, the “carbon nanofiber physical confinement - resin pyrolysis” synergistic regulation strategy provides a new idea for the structural design and performance optimization of electromagnetic wave absorbing materials.

## Resource availability

### Lead contact

Further information and requests for resources and reagents should be directed to and will be fulfilled by the lead contact, Bin Quan (binquan@nuist.edu.cn).

### Materials availability

This study did not generate new unique reagents.

### Data and code availability

• Data: Data reported in this article will be shared by the lead contact upon request.
• Code: This article does not report original code.
• Additional information: Any additional information required to reanalyze the data reported in this article is available from the lead contact upon request.

## Acknowledgments

The authors are grateful for financial support from the 10.13039/501100001809National Nature Science Foundation of China (No. 52101175), the 10.13039/501100004608Natural Science Foundation of Jiangsu Province (No. BK20210653), Opening Project Fund of Materials Service Safety Assessment Facilities (No. MSAF-2023-101), Open Project of Jiangsu Key Laboratory of New Energy Devices & Interface Science (No. KFKT2025001).

## Author contributions

Xiying Shen: Data curation, formal analysis, investigation, software, validation, and writing-original draft. Pinshu Wang, Yu Chen, and Ruiheng Jin: Data curation, formal analysis, software, and validation. Linyu Xie and Bingling He: Data curation and validation. Xiaochi Lu: Conceptualization, funding acquisition, investigation, project administration, supervision, visualization, and writing-review and editing. You Wen: validation. Bin Quan: Conceptualization, funding acquisition, investigation, project administration, supervision, visualization, and writing-review and editing. All the authors contributed to the revision and discussion of the article.

## Declaration of interests

The authors declare no competing interests.

## STAR★Methods

### Key resources table

**Table:** 

REAGENT or RESOURCE	SOURCE	IDENTIFIER
**Chemicals, peptides, and recombinant proteins**		

Paraformaldehyde	Aladdin	CAS:30525-89-4 https://www.aladdin-e.com/zh_cn/c104188.html
Melamine	Macklin	CAS:108-78-1 https://www.macklin.cn/products/M766808
Ammonia water solution	Aladdin	CAS:1336-21-6 https://www.aladdin-e.com/zh_cn/a291784.html
Carbon fibers	ZHEJIANG YAMEI NANO TECHNOLOGY CO.,LTD	A.P.S: 20–50 nm:10-20 μm, Purity: 99.9% https://yameinano.cnpowder.com.cn/product_173711.html

**Software and algorithms**		

Origin 2021	OriginLab Corporation	https://www.originlab.com
MDI Jade 6	Materials Data Corporation	https://www.materialsdata.com/prodjd.html
CST Studio Suite 2018	Computer Simulation Technology AG	https://www.3ds.com

### Method details

#### Materials

##### Preparation of MF resin precursor

The MF resin precursor was prepared by solution polymerization. In a beaker equipped with a magnetic stirrer and heating platform, 25.0 g of paraformaldehyde was dispersed in 100 mL of deionized water. The reaction system was heated to 85°C and maintained at a constant temperature with stirring for 1 h for pre-polymerization. Subsequently, 6.30 g of melamine was added to the reaction system, and the stirring reaction was continued under stable reaction temperature conditions. The pH value of the reaction solution was slowly adjusted to 8.0 with ammonia water solution. The stirring was continued until the solution became completely clear and transparent. After the reaction was completed, the system was transferred to a freezer for pre-freezing for 12 h. Then, the pre-frozen sample was placed in a freeze dryer and freeze-dried for 48 h at a cold trap temperature of −40°C and a vacuum degree of 0.1 mbar to obtain white and fluffy melamine-formaldehyde resin precursor powder. The obtained product was sealed and stored in a desiccator for later use.

##### Preparation of MD-CFPC

The porous carbon material MD-CFPC was prepared by solvent-assisted pyrolysis. The specific steps are as follows: accurately weigh 1 g of MF precursor in a beaker, and add 50 mg (MD-CFPC1), 100 mg (MD-CFPC2), and 250 mg (MD-CFPC3) of CFs respectively according to the experimental design (the sample without CFs is recorded as MD-CFPC0). Add an appropriate amount of deionized water to form a homogeneous slurry, then stir for 30 min to ensure thorough mixing of the components. Transfer the mixture to a vacuum drying oven and dry at 60°C for 12 h to obtain the precursor block. An appropriate amount of block is loaded into a quartz boat and placed in a tube furnace, and subjected to programmed temperature carbonization in a nitrogen atmosphere (heating at a rate of 2°C/min to 700°C and holding for 5 h), and finally obtain the target material. In this work, 700°C was adopted as the carbonization temperature to prepare nitrogen-doped amorphous carbon using MF resin. By taking advantage of its moderate dielectric constant characteristics and forming a heterogeneous system with graphitic carbon fibers, the electromagnetic parameters were optimized and regulated, thereby enhancing the microwave absorption performance.

#### Characterization

The X-ray diffraction (XRD) patterns were detected by D/teX Ultra 250 detectors (Rigaku Co.) with Cu Kα (λ = 1.5406 Å) radiation. The microstructure of the sample was examined using scanning electron microscope (SEM, ZEISS GeminiSEM 300) and transmission electron microscope (TEM, FEI Talos F200X G2). Raman spectroscopy was carried out using a WITec alpha300 R Raman spectrometer. The elemental composition and valence states of the samples were characterized using X-ray photoelectron spectroscopy (XPS, Thermo Scientific K-Alpha). The EM parameters were recorded at 2–18 GHz by coaxial method using vector network analyzer (VNA, Agilent PNA N5224A). In details, the testing samples with standard toroidal shape (inner diameter of 3.04 mm, outer diameter of 7.00 mm) were fabricated with paraffin. According to the transmission line theorem, the reflection loss (RL) of the material can be determined by using the following formula^48^:

(Equation 3) $$Z_{in}=Z_{0}\sqrt{\frac{\mu_{r}}{\varepsilon_{r}}}\tan\;h\left(j\frac{2\pi fd}{c}\sqrt{\varepsilon_{r}\mu_{r}}\right)$$

(Equation 4) $$RL\left(dB\right)=20\;\log\left|\frac{Z_{in}-Z_{0}}{Z_{in}+Z_{0}}\right|$$

Where, Z_0_ and Z_in_ represent the characteristic impedance of free space and input characteristic impedance respectively; ε_r_ and μ_r_ denotes the permittivity and permeability respectively.

### Quantification and statistical analysis

Graphs in the main text and supplementary files were generated from the raw data using Origin 2021. XRD was analyzed using MDI Jade 6. Radar Cross Section (RCS) was generated using CST Studio Suite 2018 simulations.
