# Supplementary material for: Carbon nanofiber-reinforced 3D porous aerogels for tunable low-frequency electromagnetic wave absorption
Source: iScience. 2025 Nov 26;28(12):114224. doi: 10.1016/j.isci.2025.114224 (PMC12723370; doi:10.1016/j.isci.2025.114224)
Supplement: Document S1. Figures S1–S5 and Table S1 [file mmc1.pdf]

iScience, Volume *28*

Supplemental information

Carbon nanofiber-reinforced 3D porous aerogels for tunable low-frequency electromagnetic wave absorption

Xiying Shen, Pinshu Wang, Yu Chen, Ruiheng Jin, Linyu Xie, Binglin He, Xiaochi Lu, You Wen, and Bin Quan

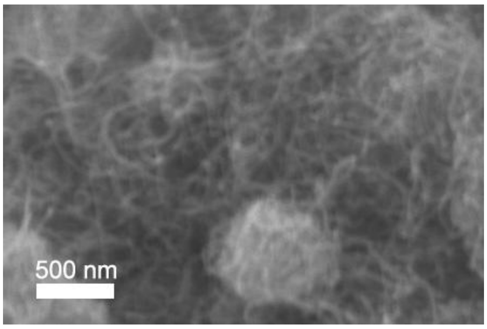

Figure S1. The SEM image of CF.

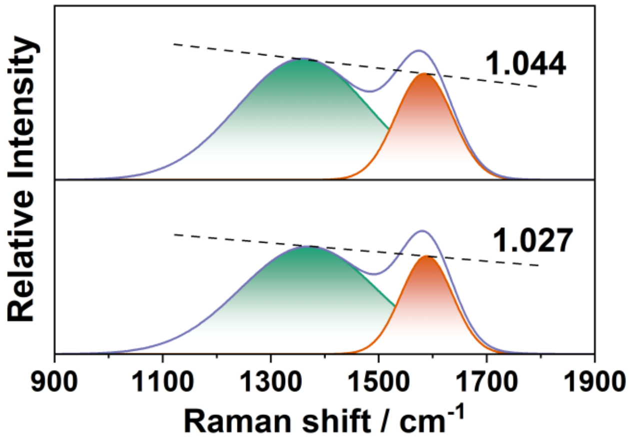

Figure S2. Raman spectra of MD-CFPC0 and MD-CFPC2.

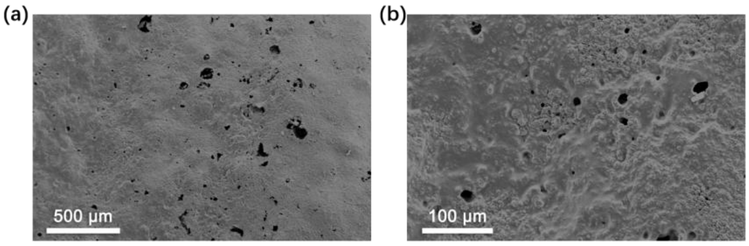

Figure S3. SEM images of MD-CFPC3.

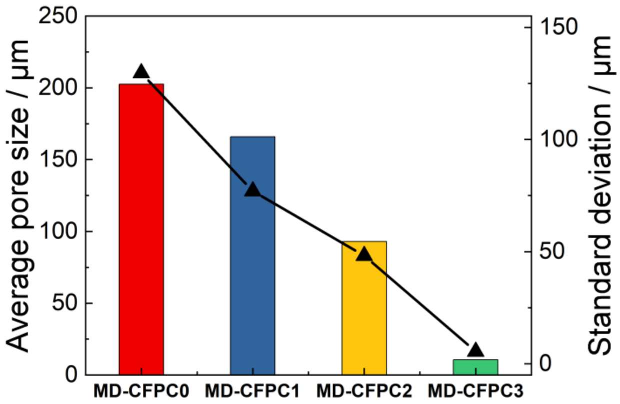

Figure S4. The average pore size in the SEM image and the standard deviation of the pore size.

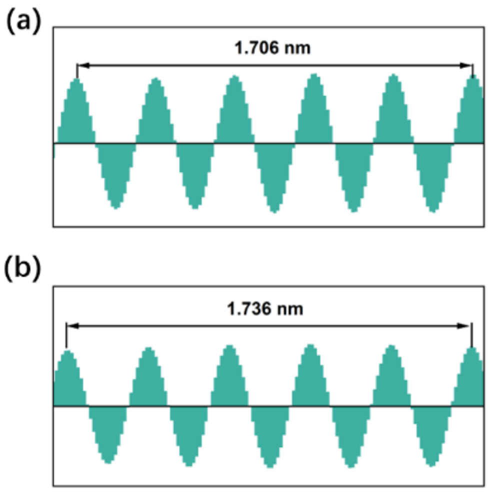

Figure S5. Lattice spacing corresponding to the high-resolution TEM images of MD-CFPC2.

Table T1 Comparison of absorption properties of different CF composite materials.

Sample	Frequency (RL_{min}) / GHz	RL_{min}/dB	References
$CoCrFeO_4$/CF	9.25	-24.10	1
CoNi/CF	17.10	-55.40	2
Polyurethane/CF	13.20	-11.01	3
Modified CF	15.10	-46.20	4
Fe_3O_4/CF@Fe_3O_4/rGO	10.80	-52.50	5
ceramic–based CF	13.20	-20.40	6
CoO_x /CFs	13.41	-45.16	7
N-doped porous CFs	9.28	-51.05	8
MD-CFPC	6.26	-42.38	This work

ADDITIONAL RESOURCES

1. Cheng, J., Liu, J., Cui, B., and Li, Y. (2025). Preparation and wave-absorbing properties of cobalt-chromium ferrite/carbon fiber composites. J. Mater. Sci.: Mater. Electron. *36*, 273. 10.1007/s10854-025-14322-z.
2. Jin, D., Yang, X., and Wei, Y. (2022). Preparation and enhancement microwave absorption properties of carbon fibers coated with CoNi alloy by solvothermal. J. Mater. Sci.: Mater. Electron. *33*, 4510–4522. 10.1007/s10854-021-07641-4.
3. Ban, G., Liu, Z., Ye, S., Yang, H., Tao, R., and Luo, P. (2017). Microwave absorption properties of carbon fiber radar absorbing coatings prepared by water-based technologies. RSC Adv. *7*, 26658–26664. 10.1039/C7RA02631E.
4. Li, X., Liu, S., Meng, X., Zhang, S., Yu, M., Wu, L., and Liang, X. (2024). Modified antistatic carbonaceous fiber with excellent hydrophobicity, environmental stability and radar

absorption performance. Carbon *229*, 119501. 10.1016/j.carbon.2024.119501.
5. Gang, Q., Niaz Akhtar, M., and Boudaghi, R. (2021). Development of high-efficient double layer microwave absorber based on Fe3O4/carbon fiber and Fe3O4/rGO. J. Magn. Magn. Mater. *537*, 168181. 10.1016/j.jmmm.2021.168181.
6. Li, X., Zhu, L., Su, Z., Li, X., Yu, W., and Zou, B. (2024). Design and properties of ceramic–based microwave absorbing composites with carbon fibres as absorber. Ceram. Int. *50*, 6836–6844. 10.1016/j.ceramint.2023.12.027.
7. Liu, Y., Zhang, Z., Xiao, S., Qiang, C., Tian, L., and Xu, J. (2011). Preparation and properties of cobalt oxides coated carbon fibers as microwave-absorbing materials. Appl. Surf. Sci. *257*, 7678–7683. 10.1016/j.apsusc.2011.04.007.
8. Guo, M., Lin, M., Xu, J., Pan, Y., Ma, C., and Chen, G. (2024). Reduced graphene oxide modified nitrogen-doped chitosan carbon fiber with excellent electromagnetic wave absorbing performance. Nanomaterials *14*, 587. 10.3390/nano14070587.
